# New species and faunistic records of Chironomidae (Diptera, Insecta) from Belize

**DOI:** 10.3897/zookeys.1270.178431

**Published:** 2026-02-25

**Authors:** Armin Namayandeh, Adrian A. Vasquez

**Affiliations:** 1 Department of Environmental and Life Sciences, Trent University, 1600 West Bank Drive, Peterborough, ON, Canada Trent University Peterborough Canada; 2 Department of Biology, College of Liberal Arts and Sciences, Mercer University, 1501 Mercer University Drive, Macon, GA 31207, USA Mercer University Macon United States of America

**Keywords:** Belize, Belize River, Chironomidae, faunistic records, Guanacaste National Park, new species

## Abstract

Four new species and 11 new faunistic records of Chironomidae are described and reported from the Guanacaste National Park, a small patch of tropical forest in the Belize Lowlands, adjacent to the Belize River and in proximity to an urban area. *Labrundinia belizensis***sp. nov**. has a unique pattern and coloration on its abdominal tergites and possesses a transverse sternapodeme without an anterior process, distinguishing it from other related species. *Cricotopus mayaorum***sp. nov**., and *Lauterborniella solangelhugo***sp. nov**. are unusual in their characteristics of antenna, thorax, and hypopygium in comparison to the related species in their respective genus. The Polypedilum (Tripodura) francisae**sp. nov**. can be distinguished from its congeners by a broad and conoid anal point and a bulbous superior volsella that has a basolateral brush of long spine-like setae. Additionally, in this study, we report on new faunistic records of Chironomidae with both the Nearctic and the Neotropical influence. Given the short period of sampling (i.e., a few days) in which this study was conducted and the number of discoveries, the importance of the Neotropical study of Chironomidae biodiversity can be recognized.

## Introduction

To date, only a handful of studies have been conducted on the Chironomidae of Belize. In general, the freshwater macroinvertebrates of Belize are poorly studied, without a comprehensive countrywide study of the major taxonomic groups (Carrie and Kay 2014). Possibly the earliest study on Chironomidae of Belize is the work of Bretschko et al. (1982), who investigated and reported the larvae of the genus *Pontomyia* Edwards, 1926, inhabiting the fore reef of Carrie Bow Cay in the Caribbean Sea. Based on the exuviae of female and pupa, Bretschko et al. (1982) suggested that the species is likely that of *Pontomyia natans* Edwards, 1926. Spies and Reiss (1996) listed only three named species of Chironomidae from Belize: *Nanocladius bubrachiatus* Epler, 1986, and *Ablabesmyia cinctipes* (Johannsen, 1946). The record of *N. bubrachiatus* was obtained through the collection of W. L. Shepard, and its determination by M. Spies. It was not part of the original description of the species from Honduras by Epler (1986). The origin of the record for *A. cinctipes* is unclear. Other records of the described species from Belize are from the Yucatán Peninsula. The peninsula encompasses northern Belize, northern Guatemala (Peten area), and southeast Mexico. Vinogradova (2008) described four new Polypedilum (Tripodura) spiesi, Polypedilum (Tripodura) scharfi, Polypedilum (Tripodura) nazarovae, and *Polypedilum rohneri* from the Belizean region of the Yucatán Peninsula. Vinogradova et al. (2009) described two *Tanytarsus* species: *Tanytarsus poqomchi* Vinogradova, Riss & Spies, 2009 and *Tanytarsus kiche* Vinogradova, Riss & Spies, 2009. Our online search yielded only one other study in this region, Pérez et al. (2013), which listed four named species in addition to 62 unknown species across 55 genera from the Yucatán Peninsula: *Chironomus anthracinus* Zetterstedt, 1860, *Chironomus plumosus* Linnaeus, 1758, *Corynocera ambigua* Zetterstedt, 1838, and *Parakiefferiella fennica* Tuiskunen, 1986. Some of these records may require further confirmation. However, what can be extracted from their study is that a larger number of species were inhabiting the Yucatán Peninsula lowlands compared to other regions, suggesting that chironomids are very abundant and diverse in the low-elevation Neotropical regions (Pérez et al. 2013).

The Guanacaste National Park, where this study was conducted, is not part of the Yucatán Peninsula. It is in proximity to the transition zone between the Maya Mountain foothills or the uplands and the coastal plains, the true lowlands, and is found adjacent to the Belize River. However, it is still considered a part of the lowland forest ecoregion (see Latha et al. 2016). The northern lowlands of Belize are characterized by broadleaf forest over limestone and pine savanna on sandy soils; wetland swamps, and low gradient, slow-flowing freshwater rivers and lagoons are also common to this region (Bridgewater 2012; Carrie and Kay 2014). The abundance and diversity of freshwaters in this region of Belize may account for the higher diversity of Chironomidae. This study was conducted over the course of a few days in Guanacaste National Park, a small patch of tropical forest in the Belize Lowlands, adjacent to the Belize River, and in proximity to an urban area. Despite the short sampling period and small area covered, four new species and 11 new faunistic records of Chironomidae were described and discovered from this ecosystem. Our discoveries highlight the potential for further exploration of this area with continued research into the region’s Chironomidae fauna.

In this study, we have described the following newly discovered species: *Labrundinia belizensis* sp. nov. is distinguished from other related Neotropical species by the pattern and coloration of its abdominal tergites and a transverse sternapodeme without an anterior process. *Cricotopus mayaorum* sp. nov. is an unusual member of its genus that, for now, cannot be placed in any subgenera with certainty. The new species is characterized by a posterior scutum covered in numerous microtrichia and a fusiform gonostylus. *Lauterborniella solangelhugo* sp. nov. belongs to *Lauterborniella* Thienemann & Bause, 1913, based on the presence of pulvilli, fore tibia spur, separate tibial combs, and the anal tergite band. However, the unusual absence of anal points resembles the species of the genus *Apedilum* Townes, 1945. Furthermore, the species has a distinguishing high antennal ratio, not seen in other members of its genus. The Polypedilum (Tripodura) francisae sp. nov. is distinguished from related species by a broad and conoid anal point with basal notches and a bulbous superior volsella that has a basolateral brush of long spine-like setae.

## Materials and methods

### Study area

**Figure 1.**
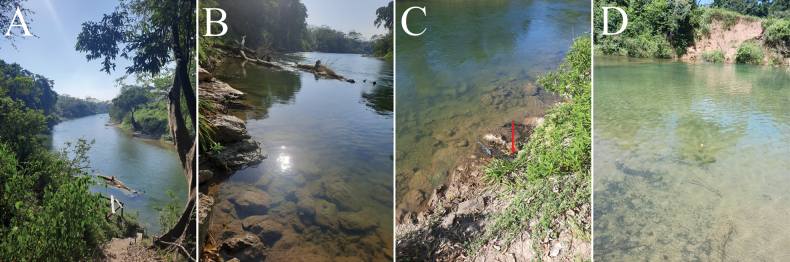
The study sites and habitats of Chironomidae collected from the Guanacaste National Park, Belize. **A**. Elevated view of the Belize River; **B**. An up-close view of the Belize River and its substrate; **C**. A small inflow into the Belize River (arrow); **D**. Roaring Creek, a tributary to the Belize River.

The study collection sites are located in Guanacaste National Park, a fifty-acre parcel of secondary growth tropical forest located on the north side of the Western Highway and part of the Belize River valley. It was once a Mayan territory, with many of their artifacts discovered in the park (Garber 2004). It was established as a National Park in 1990 by efforts of the Belize Audubon Society and is protected by the Belize government under the status of a National Park (Walker and Walker 2011; Latha et al. 2016). The name of the park originates from the giant Guanacaste trees (*Enterolobium cyclocarpum* (Jacq.) Griseb) growing in the southwestern area of the park. The park is bordered by the Belize River (17.263433, -88.786359) in the north and by Roaring Creek in the west (17.261937, -88.790266). The section of the Belize River in the Park where the collections were obtained is dominated by clay and large boulder substrates with high riparian vegetation covering the adjacent terrestrial environment (Fig. 1A, B). Numerous seeps and small tributaries flow into the river (Fig. 1C). However, the major tributary to the river is the Roaring Creek, which has a dominating clay substrate where it flows into the Belize River (Fig. 1D). Both lotic habitats have steep, carved, canyon-shaped channels with access only possible through staircases. During the rainy season, the river can rise up to 12 meters, which is probably the reason for the carved canyon-shaped channels that follow the meander of this important river (Garber 2004).

### Sampling collection, preparation, imagery, and terminology

All adult Chironomidae were collected using a handheld net (250 µm sweep net BioQuip, Rancho Dominguez, CA) by aerial sweeping or by brushing the riparian vegetation, fences near trails, and river side locations with initial sorting and selection of Chironomid flies done using a Swift M30 (Swift Microscopes, Carlsbad, CA) stereomicroscope. Collected specimens were initially stored in isopropanol and then transferred and preserved in 90% ethanol. Adult males were slide-mounted in Mount-Quick “Aqueous” Daido Sangyo Co., LTD. Japan, following the procedure outlined in Langton and Pinder (2007). The imagery of the specimens was conducted using an OMAX A3550U camera mounted on an AMScope compound microscope. Illustrations were produced in Inkscape 1.2.2 from specimen photographs (Inkscape Project 2023).

Abbreviations, morphological terminology, and measurements follow those used by Sæther (1980). The following abbreviations for the legs’ length and ratios are used: **BV** = Beinverhältnisse (fe + ti + ta_1_/sum of ta_2_–ta_5_); **fe** = femur; **LR** = Leg Ratio (metatarsus/tibia); **P_1–3_** = front, mid, and hind legs; **ta_1–5_** = tarsal segments 1 to 5; **ti** = tibia; **SV** = Schenkel-Shiene-Verhältnis (fe + ti/t_1_).

All types and voucher specimens of Chironomidae from Belize are deposited at the Albert J. Cook Arthropod Research Collection (**ARC**), Michigan State University. Additional vouchers examined are deposited at the Canadian National Collection of Insects, Arachnida, and Nematoda (**CNC**) and the personal collections of Dr. John H. Epler in Crawford, Florida (**JHE**) and Mr. Patrick Hudson in Ypsilanti, Michigan (**PLH**).

Additional materials examined for this study include: 1 male; *Stictochironomus palliatus* (Coquillet, 1902); USA, Georgia, Oconee River, Highway 46; 22.ix.1987; leg. B. A. Caldwell; Canadian National Collection (CNC) CH8949. 1 male; *Stictochironomus palliatus* (Coquillet, 1902); USA, Arkansas, Arkansas Co., White River-NWR at Jackson Bay, White River, 20.vi.2001, leg. Chordas & Chapman, dep. PLH. 2 males; *Stictochironomus palliatus* (Coquillet, 1902); USA, Kentucky, Crittenden Co., Ohio River, 23.vi.199, leg. Chordas & Stewart, dep. PLH. 1 male; Polypedilum (Tripodura) digitifer Townes, 1945); USA, Alabama, Tallapoosa Co.; Saugahatchee Creek, 1.8 miles NNE of Reeltown at Hwy 49, T.19N R.23E sec 18, 15.vi.1988; leg. W.E. Garret [APC-6]; dep. JHE. 1 male; Polypedilum (Tripodura) digitifer Townes, 1945); USA, Alabama, Elmore Co.; Tallapoosa River, 3.4 miles south of Ware (Dozier’s), T.17N R.19E sec 34, 20.iv.1988; leg. W.E. Garret [APC-2]; dep. JHE. 1 male; Polypedilum (Tripodura) digitifer Townes, 1945; USA, Alabama, Baldwin Co., Little Briar Creek, 38 84N 87 94W, 13.ix.2000; leg. J.E McCreedie; dep. JHE. 1 male; Polypedilum (Tripodura) digitifer Townes, 1945); USA; G3a; dep. JHE. 1 male; Polypedilum (Tripodura) griseopunctatum (Malloch, 1915); Sandy Creek, 2.2 miles WSW of Camp Hill at Hwy 34, T.21N R.24E sec 19; 15.vi.1988; leg. W.E. Garrett [APC-12]; dep. JHE.

The following references were used to identify the new faunistic records: Andersen and Mendes (2002a), Andersen et al. (2010, 2018), Borkent (1984), Hirvenoja (1973), Kyerematen and Andersen (2002), Reiss (1974), and Sublette and Sasa (1994).

## Results

### Faunistic records

**Figure 2.**
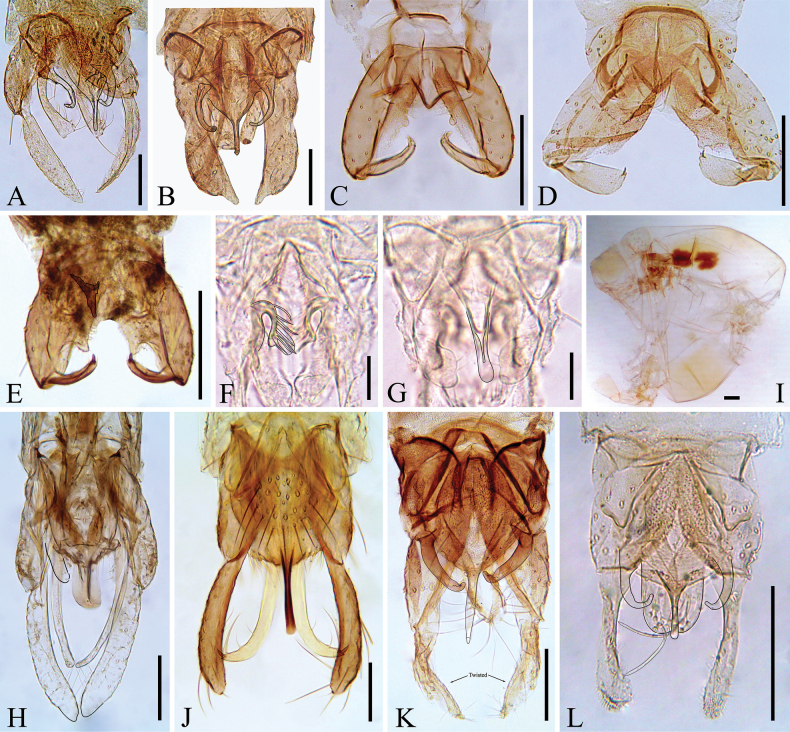
Gallery of the new faunistic records of adult males collected from Belize (**A–K**). **A**. *Endotribelos albatum* Sublette & Sasa, 1994; **B**. *Geoldichironomus fluctuans* Reiss, 1974; **C, D**. *Mesosmittia patrihortae* Sæther, 1985; **E**. *Pseudosmittia joaquimvenancioi* (Messias & Oliveira, 2000); **F, G**. *Rheotanytarsus meridionalis* (Johannsen, 1938); **H**. Stenochironomus (Petalopholeus) albidorsalis Borkent. 1984; **I, J**. Stenochironomus (Petalopholeus) quadrinotatus Borkent, 1984; **K**. *Stictochironomus palliatus* (Coquillett, 1902); **L**. *Xestochironomus ankylis* Sublette & Sasa, 1994; **A–E, H, J–L**. Hypopygium; **F**. Median volsella; **G**. Anal point and superior volsella; **I**. Thorax. *Cricotopus* (*s.s*.) bicinctus (Meigen, 1818) was omitted. Scale bars: 100 µm (**A-E, H, I**), 25 µm (**F, G**).

The following species are new faunistic records for Belize: *Axarus rogersi* (Beck & Beck, 1958); *Cricotopus* (*s.s*.) bicinctus (Meigen, 1818); *Endotribelos albatum* Sublette & Sasa, 1994; *Geoldichironomus fluctuans* Reiss, 1974; *Mesosmittia patrihortae* Sæther, 1985; *Pseudosmittia joaquimvenancioi* (Messias & Oliveira, 2000); *Rheotanytarsus meridionalis* (Johannsen, 1938); Stenochironomus (Petalopholeus) albidorsalis Borkent, 1984; Stenochironomus (Petalopholeus) quadrinotatus Borkent, 1984; *Stictochironomus palliatus* (Coquillett, 1902); *Xestochironomus ankylis* Sublette & Sasa, 1994 (Fig. 2).

The single adult male specimen of *C. bicinctus* recovered from the samples was in poor condition. As such, we did not include it in Fig. 2. The three adult males of *M. patrihortae* matched the description provided by Sæther (1985) and Andersen and Mendes (2002a). The antennal ratio of the Belizean *M. patrihortae* was 1.09–1.20, the wing length was 0.91–0.95 mm, the VR 1.33–1.37, and the costa extension 34–49 µm. Similar to what has been illustrated in fig. 4C–E by Sæther (1985) and fig. 35 by Andersen and Mendes (2002a), the gonostylus of the *M. patrihortae* species shows varying shapes and sizes (Fig. 2C, D). According to Kyerematen and Andersen (2002)*R. meridionalis* (Johannsen) is similar to *Rheotanytarsus hamatus* Sublette & Sasa, 1994. However, it can be separated from the latter based on median volsella having an apical plate (Fig. 2F). To the best of our knowledge *S. palliatus* is the first record of a named *Stictochironomus* in the Neotropical region. The gonostylus of the only specimen of *S. palliatus* collected in this study was twisted, not showing its expanded semi-circular shape. However, comparison of the Belizean specimen with those from the Nearctic confirmed its identification. The other Neotropical record of the genus is from BOLD for *Stictochironomus* sp. 1SC (Sample ID: CHIR715-19) collected from Brazil. The three adult males of *X. ankylis* collected in this study matched the description provided by Sublette and Sasa (1994), with the same coloration and hypopygium characteristics (Fig. 2K). The Belizean adult male has an AR 1.20–1.30, compared to the described Guatemalan male by Sublette and Sasa (1994) with an AR of 0.79.

### Taxonomic account

**Subfamily Tanypodinae**

#### Labrundinia belizensis sp. nov.

Taxon classificationAnimaliaDipteraChironomidae

724A05D2-1D14-5FD0-A952-E4FA4617E983

https://zoobank.org/B1C89947-A80F-4E32-B802-8F068B92E047

Fig. 3

##### Type material.

***Holotype***, • 1 male; Belize: Guanacaste National Park, Belize River; 17.262083, -88.788134; leg. A.A. Vasquez; dep. ARC. ***Paratypes***, • 2 males, same as holotype.

**Figure 3.**
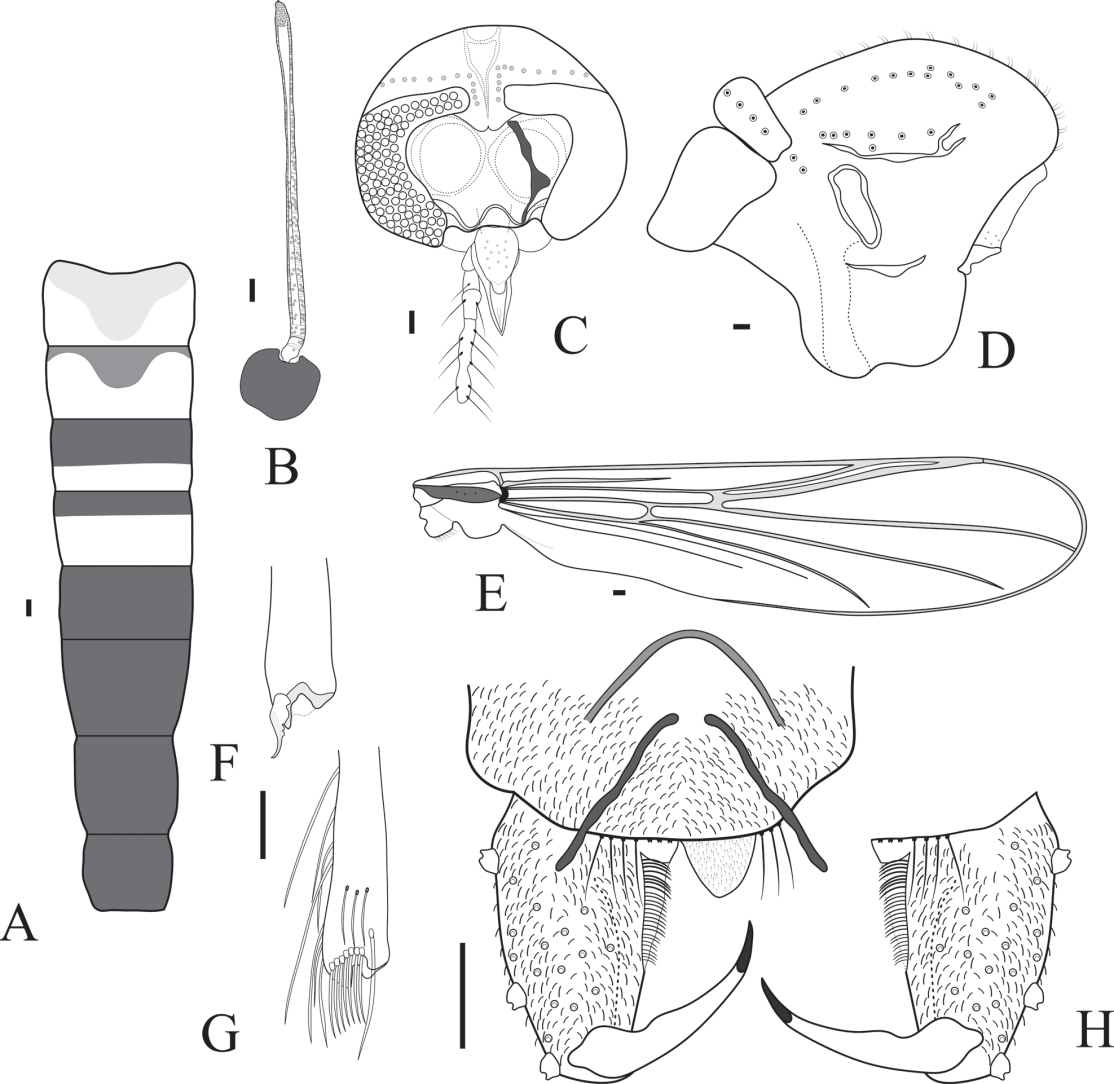
*Labrundinia belizensis* sp. nov. adult male. **A**. Abdominal tergites; **B**. Antenna; **C**. Head; **D**. Thorax; **E**. Wing; **F**. Apex of the fore tibia; **G**. Apex of hind tibia; **H**. Hypopygium. Scale bars: 25 µm.

##### Diagnostic characters.

The adult male of *Labrundinia belizensis* sp. nov. differs from related species by the combination of the following characters: Abdominal tergite I with a faintly greyish-brown mid tergite patch, tergite II with a darker and smaller mid-tergite patch; posterior 2/3^rd^ of tergite III with transverse brown band; posterior 1/3^rd^ of tergite IV with transverse brown band; tergites V–IX and hypopygium brown; AR 0.89–0.91; eye ratio 1.2–1.4; acrostichals 26; transverse sternapodeme without anterior process.

##### Description.

Male (*n* = 3; unless otherwise stated). Total length 1.79–2.08, 1.90 mm. Wing length 1.20–1.27, 1.24 mm. Total length/wing length 1.40–1.53, 1.47. Wing length/profemur length 4.08–4.77, 4.43.

***Coloration***. Head, thorax brown. Abdominal tergite I with a faintly greyish-brown mid-tergite patch, tergite II with a darker and smaller mid-tergite patch; posterior 2/3^rd^ of tergite III with a transverse brown band; posterior 1/3^rd^ of tergite IV with a transverse brown band; tergites and sternites V–IX and hypopygium brown (Fig. 3A). Sternites I–III light. Wings greyish brown. Halteres light. Femurs of all legs brown; tibia of all legs with anterior and posterior brown bands.

***Head*** (Fig. 3B, C). Antenna last flagellomere 258–271, 265 μm long; apical nipple with numerous short microtrichia and 6 sensilla chaetica (Fig. 3B); AR 0.89–0.91, 0.90 (*n* = 2); subapical setae missing. Temporal setae 12, including 2 outer verticals and 6 inner verticals, horizontally uniserial, and 4 postorbitals, vertically uniserial (Fig. 3B). Eye ratio 1.2–1.4 (2). Tentorium 103–133, 119 μm long. Clypeus 75–92, 84 μm long and 52–64, 60 μm wide (at widest section), bearing 9–15, 12 setae. Cibarial pump 179–180 μm long. Palpomeres 4 and 5 missing in all specimens examined; lengths of palpomeres 1–3 (in μm): 38–41, 40; 37–44, 41; 101–111, 106.

***Thorax*** (Fig. 3D). Acrostichals 30–34, 32, biserial, starting close to antepronotum reaching near scutellum; dorsocentrals 10–13, 12, irregularly uniserial; prealars 7, stretching close to parapsidal suture; supraalars 2; scutellars 8, uniserial. Antepronotal lobe with 5 basolateral setae.

***Wing*** (Fig. 3E). Anal lobe reduced. Costal 1.0–1.07 mm long. Distance from vein r-m to arculus 447–466, 456 μm long; R with 24–30, 27 setae; R_1_ with 19–27, 23 setae; R_4+5_ with 52–64, 58 setae. Brachiolum with 3–4 setae. Squama with 8 setae. VR 0.66–0.69, 0.67.

***Legs*** (Fig. 3F, G). All tarsal segments missing. For and mid tibia spurs small, with 2 minute inner teeth, 16–20, 18 μm long. Hind tibia spur absent; comb with 7 spines and several dorsolateral long bristles. Fore to hind tibia width (in μm) at apex as follows: 27–30, 29; 24–27, 25; 34–35. Lengths and proportions of leg segments as in Table 1.

**Table 1.** Male leg lengths (μm) and proportions of *Labrundinia belizensis* sp. nov.

	fe	ti	ta_1_	ta_2_	ta_3_	ta_4_	ta_5_	LR	BV	SV
P_1_	385–451, 424	348–378, 358	–	–	–	–	–	–	–	–
P_2_	601–653, 620	439–507, 471	–	–	–	–	–	–	–	–
P_3_	497–523, 509	564–570, 567	–	–	–	–	–	–	–	–

***Hypopygium*** (Fig. 3H). Tergite IX with 10–12 anterior setae. Membranous anal point dome-shaped, 27 μm long. Transverse sternapodeme 34–52, 42 μm long, without anterior process. Phallapodeme 51–52 μm long. Gonocoxite 85–103, 92 μm long. GcR 2.0–2.2. Gonostylus narrowing apically, 50–57, 54 μm long; megaseta 10–13, 11 μm long. HR 1.56–1.80, 1.69. HV 3.17–3.70, 3.51.

##### Etymology.

The new species is named after the country of Belize, where it was collected. The suffix -*ensis* is Latin, and it denotes belonging to a location.

##### Remarks.

The coloration of the abdominal tergite of the new species resembles that of *Labrundinia costaricae* Silva, 2014, *Labrundinia johannseni* Beck & Beck, 1966, and *Labrundinia neopilosella* Beck & Beck, 1966. The pattern of the brown bands of the new species differs on tergites I and II from that of the latter species. The new species also differs from the three related species by a higher eye ratio, a lower number of acrostichals, and a complete lack of the anterior process of the sternapodeme. It also differs from *L. johannseni* and *L. neopilosella* by a lower AR, and it differs from *L. neopilosella* by having an anal point without a notch (see da Silva et al. 2014).

###### Subfamily Orthocladiinae

#### Cricotopus mayaorum sp. nov.

Taxon classificationAnimaliaDipteraChironomidae

1FDAAE1D-95EB-52A8-887F-A1A7B2FA0629

https://zoobank.org/59348F7A-43A9-4633-AD2E-EB8F824E58CC

Fig. 4

##### Type material.

***Holotype*** • 1 male; Belize: Guanacaste National Park, Belize River; 17.263433, -88.786359; leg. A.A. Vasquez; dep. ARC.

##### Diagnostic characters.

The adult male of *C. mayaorum* sp. nov. can be separated from related species by the combination of the following characters: Microtrichia of posterior scutum numerous and long, giving the appearance of acrostichals; abdominal tergites I, VII–VIII brown. Tergites II–III with pale band on posterior 1/3 and brown band on anterior 2/3. Tergites IV–VI with pale band on the posterior 1/2 and brown band on the anterior 1/2; femurs of all legs brown; posterior 1/5 and anterior 2/5 of fore and mid tibia brown, the mid 3/5 light; the posterior 2/3^rd^ of hind tibia brown, remainder light brown; all tarsi light brown; temporal setae 4; gonostylus fusiform, without crista dorsalis.

##### Description.

Male (*n* = 1). Total length 3.21 mm.

**Figure 4.**
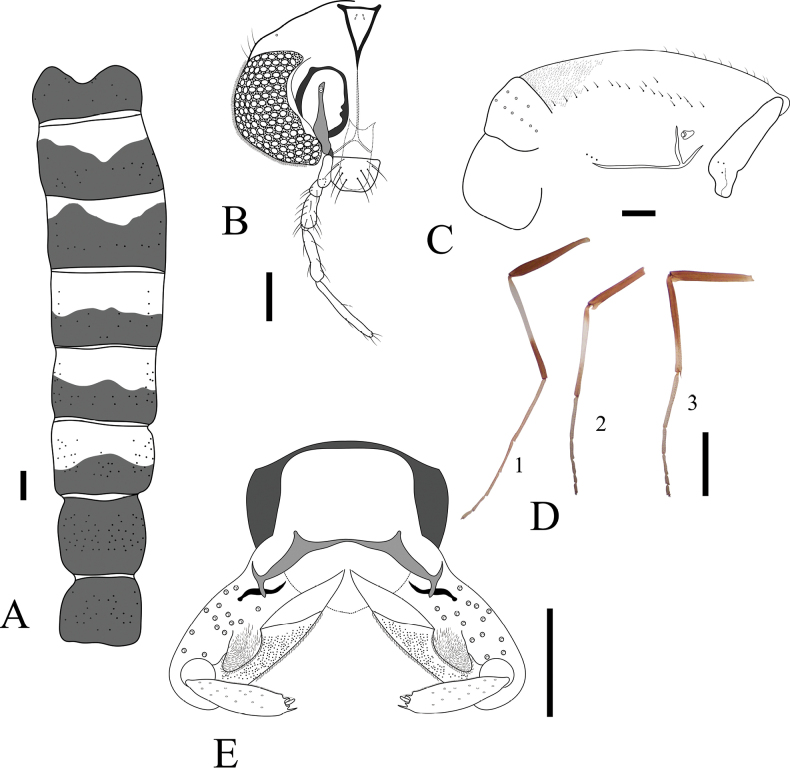
*Cricotopus mayaorum* sp. nov. (**A, B**). **A**. Abdominal tergites; **B**. Head; **C**. Thorax; **D**. Legs, fore leg (1), mid leg (2), hind leg (3); **E**. Hypopygium. Scale bars: 100 µm (**A–C**), 500 µm (**D**).

***Coloration***. Head, including antenna brown. Thorax and halteres brown. Abdominal tergites I, VII–VIII brown. Tergites II–III with light band on posterior 1/3^rd^ and brown band on anterior 2/3^rd^. Tergites IV–VI with light band on the posterior half and brown band on the anterior half (Fig. 4A). Femurs of all legs brown; posterior 1/5^th^ and anterior 2/5^th^ of fore and mid tibia brown, the mid 3/5^th^ light; the posterior 2/3^rd^ of hind tibia brown, remainder light brown; all tarsi light brown (Fig. 4D).

***Head*** (Fig. 4B). Antenna with 13 flagellomere; last flagellomere with 6 sensilla chaetica, flagellomere 2 and 3 each with a pair of long thin sensilla chaetica; AR 1.27. Eyes hairy, microtrichia surpassing the ommatidia, with short wedge-shaped dorsomedial extension. Temporal setae 4, including 3 outer verticals and 1 inner vertical. Clypeus rectangular, 78 μm long and 109 μm wide, bearing 10 setae. Tentorium 171 μm long. Length of palpomeres (in μm): 60, 46, 71, 116, 173.

***Thorax*** (Fig. 4C). Acrostichals 10; dorsocentrals 19, irregularly uniserial; prealars 3; scutellars 18, biserial. Antepronotal lobe with 3 basolateral setae. Humeral pit present. Microtrichia of the posterior scutum numerous and long, giving the appearance of acrostichals.

***Wing***. Missing.

***Legs*** (Fig. 4D). Femur of mid and hind legs with keel. Fore tibia spur missing. Mid tibial spurs 28 and 25 μm long. Hind tibial spurs 61 and 24 μm long. Hind comb with 14 spines. Lengths and proportions of legs as in Table 2.

**Table 2.** Male leg lengths (μm) and proportions of *Cricotopus mayaorum* sp. nov.

	fe	ti	ta_1_	ta_2_	ta_3_	ta_4_	ta_5_	LR	BV	SV
P_1_	728	917	589	286	226	168	96	0.6	2.9	2.8
P_2_	713	743	362	174	121	77	75	0.5	4.1	4.0
P_3_	664	778	451	225	158	88	93	0.6	3.4	3.2

***Hypopygium*** (Fig. 4E). Transverse sternapodeme 119 μm long, with well-developed oral projections. Phallapodeme 47 μm long. Superior volsella absent. Inferior volsella, a large, bent finger-like lobe, located on the mid-gonocoxite. Gonocoxite 182 μm long. Gonostylus 99 μm long, fusiform, without crista dorsalis; mega seta 12 μm long. HR 1.83; HV 3.23.

##### Etymology.

The new species is named after the Mayans, the aborigines and original inhabitants of this region. The suffix -*orum* is Latin, and it denotes belonging to multiple people.

##### Remarks.

*C. mayaorum* sp. nov. is a very unusual *Cricotopus* that cannot be easily placed in any of the known *Cricotopus* subgenera. Its hypopygium resembles species in the genus *Eukiefferiella* Thienemann, 1926, particularly *Eukiefferiella ilkleyensis* (Edwards, 1929). A combination of decumbent dorsocentrals and hairy eyes identifies this adult male as a *Cricotopus*. Additionally, the color contrast of the abdominal tergites and those of the legs separates this adult male from those of *Eukiefferiella* species. The absence of superior volsella may suggest that the species belongs to the subgenus *Cricotopus* v. d. Wulp, 1874. The simple inferior volsella may suggest it belongs to the *Isocladius* Kieffer, 1909. The presence of a thoracic humeral pit, finger-like, curved posteriorly inferior volsella, with a well-developed oral projection of sternapodeme and a reduced superior volsella suggests that this species belongs to the subgenus *Paratrichocladius* Santos Abreu, 1918. However, the dorsocentrals of the new species are short and decumbent and arise directly from the cuticle and not the pale area, which does not fit the usual description of this subgenus (see Cranston and Krosch 2015). A combination of numerous long microtrichia in the posterior of the scutum, with a fusiform gonostylus, lacking crista dorsalis, suggests that the new species may belong to a new subgenus altogether. In the absence of more materials and immature stages for comparison, it is not possible to establish this. Therefore, for now, we recognize the new species as unplaced within the genus *Cricotopus* until more specimens are obtained for molecular analysis and morphological examination, including the immature stages.

###### Subfamily Chironominae

#### Axarus rogersi

Taxon classificationAnimaliaDipteraChironomidae

(Beck & Beck, 1958)

EAECE1A3-FE71-53B4-94B0-E4800B570995

Fig. 5

##### Material examined.

1 Male; Belize, Guanacaste National Park, Belize River; 17.263433, -88.786359; leg. A.A. Vasquez; dep. ARC.

##### Diagnostic characters.

The adult male of *A. rogersi* can be separated from related species by the combination of the following characters: Abdominal tergites I, VIII, IX, and hypopygium brown; tergites II–VII with posterior 2/3^rd^ yellow and anterior 1/3^rd^ brown; AR 2.7; temporal setae 22; acrostichals 14; dorsocentrals 6; anal lobe of the wing short; lateral projection of tergite IX setose, long, narrow, and curved; basal section posteriorly sclerotized, setose and with short spines, tip well-sclerotized with inverse Y-shaped dorsal crest; superior volsella tapering with around 6 long simple setae on its ventral surface; inferior volsella digitiform and long.

##### Description.

Male (*n* = 1). Total length 5.43 mm. Wing length ~2.41 mm. Total length/wing length ~ 2.30. Wing length/profemur length ~ 1.80.

**Figure 5.**
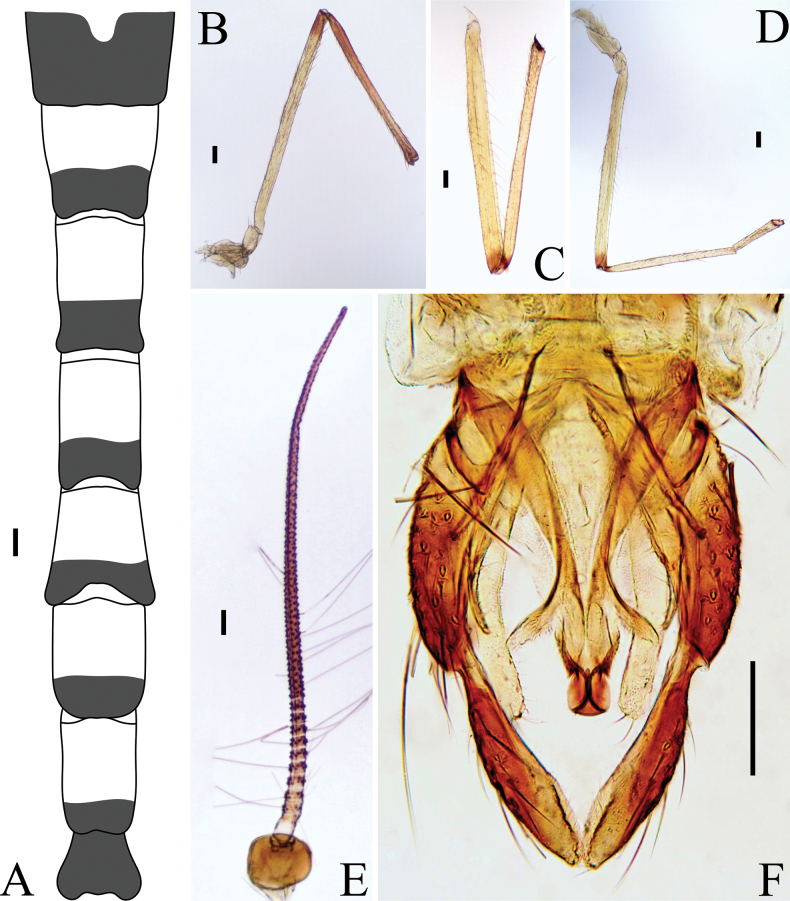
*Axarus rogersi* (Beck & Beck, 1958) adult male. **A**. Abdominal tergites; **B**. Fore femur and tibia; **C**. Mid femur and tibia; **D**. Hind femur and tibia; **E**. Antenna; **F**. Hypopygium. Scale bars: 50 µm.

***Coloration***. Head, including antenna brown. Thorax yellowish brown with paler coloration in scutellar and preepisternal region, and brown streaks at apical portion of the anterior scutum and postnotum. Abdominal tergites I, VIII, IX, and hypopygium brown. Tergites II–VII with posterior 2/3^rd^ yellow and anterior 1/3^rd^ brown (Fig. 5A). Abdominal sternites yellow. Wings greyish-brown; halteres yellow. Anterior 1/10^th^ of the fore femur and entire tibia brown, posterior 9/10^th^ of the fore femur yellow; anterior 1/10^th^ and posterior 1/10^th^ of the mid and hind fore femur brown, remainder of mid and hind femur and tibia yellow (Fig. 5B–D).

***Head***. Antenna with 11 flagellomere, 2^nd^ and 3^rd^ flagellomere with pair of long and slender sensilla chaetica, each twice the size of the segment bearing them; AR 2.67 (Fig. 5E). Temporal setae 22, uniserial, including 13 postorbitals, 3 outer verticals, and 6 postoculars. Clypeus rectangular, 58 μm long and 75 μm wide, bearing 22 setae. Tentorium 115 μm long. Third palpomere missing; length of remaining palpomere (in μm): 24, 20, 101, 116.

***Thorax***. Acrostichals 14; dorsocentrals 6, uniserial and widely distributed; prealars 6; scutellars 8, uniserial. Antepronotal lobe with 2 basolateral setae.

***Wing***. Damaged. Anal lobe developed, though short. R with 37 setae; R_1_ with 25 setae; R_4+5_ with 26 setae. Brachiolum with 2 setae. Squama with 5 setae. Microtrichia visible at 10 × magnification.

***Legs***. Tarsal segments of all legs missing. Fore tibia with scale and no spur. Mid tibia spurs 29 and 17 μm long. Hind tibia spurs 31 and 29 μm long. Lengths and proportions of legs as in Table 3.

**Table 3.** Male leg lengths (μm) and proportions of *Axarus rogersi* (Beck & Beck, 1958).

	fe	ti	ta_1_	ta_2_	ta_3_	ta_4_	ta_5_	LR	BV	SV
P_1_	1350	1050	–	–	–	–	–	–	–	–
P_2_	1322	1226	–	–	–	–	–	–	–	–
P_3_	1535	1375	–	–	–	–	–	–	–	–

***Hypopygium*** (Fig. 5F). Lateral projection of tergite IX setose, long, narrow, and curved, 62 μm long. Tergite band thick anteriorly. Anal point 118 μm long and 55 μm wide at its widest point; basal section posteriorly sclerotized, setose and with short spines, tip well-sclerotized with inverse Y-shaped dorsal crest. Transverse sternapodeme 86 μm long, without oral projections. Phallapodeme 162 μm long. Gonocoxite 194 μm long. Superior volsella tapering 127 μm long, with around 6 long simple setae on its ventral surface. Inferior volsella digitiform, 272 μm long 36 μm wide at base. Gonostylus 202 μm long. HR 0.96 HV 2.69.

##### Remarks.

*Axarus rogersi* is a well-known species of *Axarus* Roback, 1980, reported from the Neotropical region, Costa Rica, Mexico, Nicaragua, and the Nearctic, Florida, United States (Andersen et al. 2018; Andersen and Mendes 2002b; Bello-González et al. 2024; Oliver et al. 1990). Beck and Beck (1958) only briefly described the species characteristics. In this study, we provided a detailed description and measurements of the species based on a single male specimen we collected. Although this does not provide a range for its characteristics; however, it provides a basis from which future measurements can be compared.

#### Lauterborniella solangelhugo sp. nov.

Taxon classificationAnimaliaDipteraChironomidae

2C6039D2-D375-5E6E-9F7B-85A645FF50C9

https://zoobank.org/984DD108-80F6-4AC5-B372-80F23C0801DC

Fig. 6

##### Type material.

***Holotype*** • 1 male; Belize: Guanacaste National Park, Roaring Creek, trib. to Belize River; 17.261937, -88.790266; leg. A.A. Vasquez; dep. ARC. ***Paratypes*** • 2 males; same as holotype.

##### Diagnostic characters.

The adult male of *L. solangelhugo* sp. nov. can be separated from the related species by a combination of the following: Abdominal tergites I–IV and VI–VIII brown with a narrow and pale apical transverse band; tergite V uniformly light; AR 1.2–1.3; temporal setae 10, uniserial; acrostichals 5–8; dorsocentrals 8–12; R + R_1_ with 16–26; R_4+5_ with 12–16; VR 1.2–1.4; mid tergite IX with 5 or 6 long, simple setae, apex of tergite IX with rows of short, simple setae; anal point absent; sternapodeme tick, without oral projections.

##### Description.

Male (*n* = 3; unless otherwise stated). Total length 2.81–3.42, 3.21 mm. Wing length 1.48–1.62, 1.56 mm. Total length/wing length 1.89–2.16, 2.05. Wing length/profemur length 1.48–1.52, 1.49.

**Figure 6.**
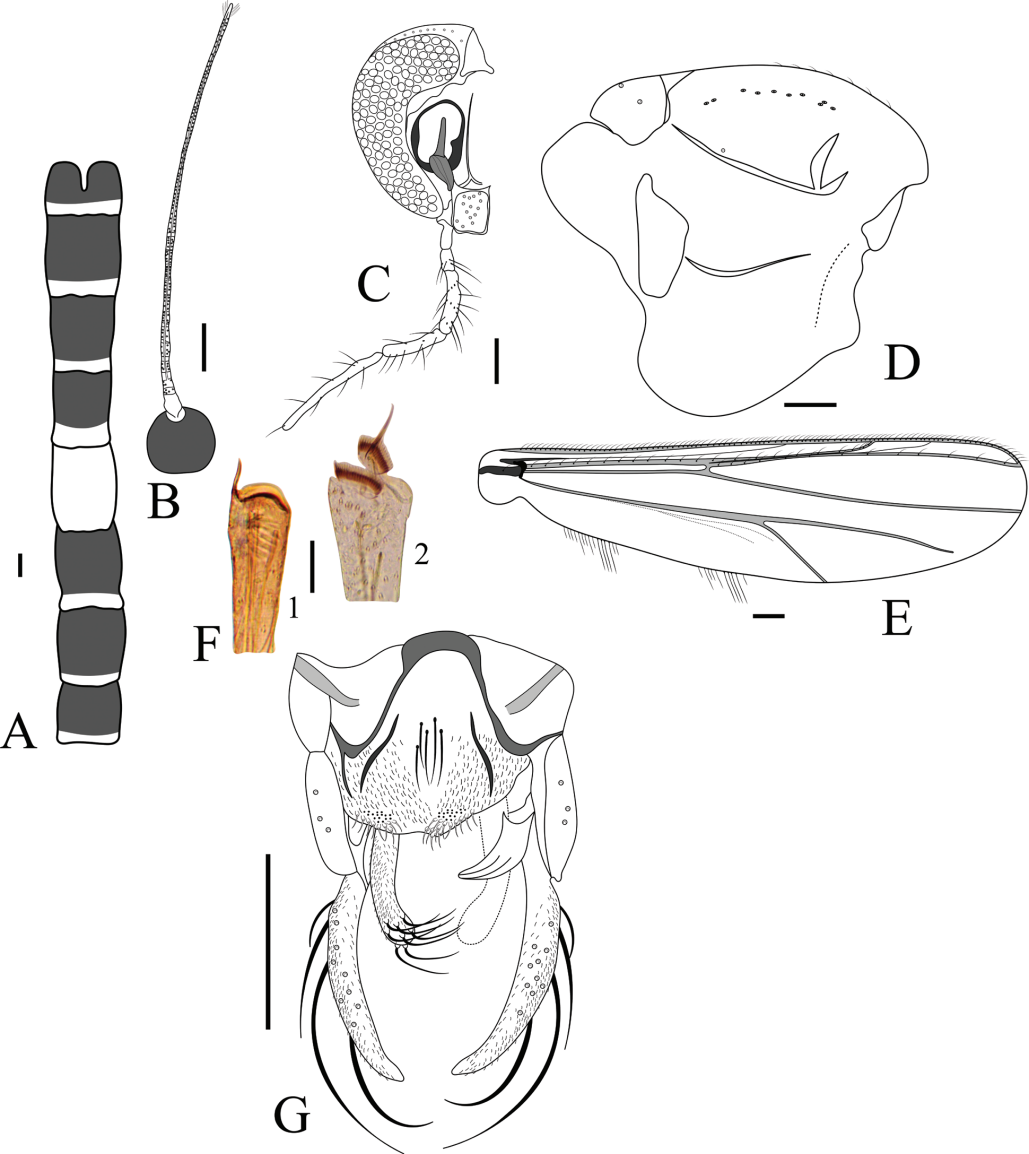
*Lauterborniella solangelhugo* sp. nov. adult male. **A**. Abdominal tergites; **B**. Antenna; **C**. Head; **D**. Thorax; **E**. Wing; **F**. Apex of fore tibia (1), apex of mid tibia (2); **G**. Hypopygium. Scale bars: 100 µm.

***Coloration***. Head, antenna, and thorax brown. Wing yellowish-brown. Halteres light. Anterior 1/4^th^ of fore femur and anterior 1/3^rd^ of fore tibia brown, remainder of segments stramineous; other legs stramineous. Abdominal tergites I–IV and VI–VIII brown with a narrow and pale apical transverse band; tergite V uniformly light (Fig. 6A); abdominal sternites I–V stramineous, VI–VIII brown.

***Head*** (Fig. 6B, C). Antenna with 13 flagellomeres, flagellomeres 2–4 with a pair of long and slender sensilla chaetica, last flagellomere with 6 sensilla chaetica; AR 1.21–1.27, 1.24. Temporal setae 10, uniserial (*n* = 2). Clypeus nearly squared, 73–87, 81 μm long and 68–87, 76 μm wide, bearing 10–14, 12 setae. Tentorium 125–166, 148 μm long. Length of palpomeres (in μm): 45–65, 56; 40–44, 42; 112–123, 118; 122–141, 132; 266 (*n* = 1).

***Thorax*** (Fig. 6D). Acrostichals 5–8, 7 (*n* = 2); dorsocentrals 8–12, 10, uniserial; prealars 1; scutellars 4, uniserial. Antepronotal lobe well-separated and bare.

***Wing*** (Fig. 6E). Anal lobe well-reduced. R + R_1_ with 16–26, 22 setae; R_4+5_ with 12–16, 13 setae. Brachiolum with 1 seta. VR 1.24–1.36, 1.29. Squama bare. Microtrichia visible at 20 × magnification.

***Legs*** (Fig. 6F). Fore tarsus missing. Fore tibia with shallow scale and single spur, 29–33, 32 (Fig. 6F_1_). Mid tibia with divided comb and single spur, 43–58, 50 μm long (Fig. 6F_2_). Hind tibia with divided comb and single spur, 57–65, 61 μm long. Pulvilli present. Lengths and proportions of legs as in Table 4.

**Table 4.** Male leg lengths (μm) and proportions of *Lauterborniella solangelhugo* sp. nov.

	fe	ti	ta_1_ (*n* = 1)	ta_2_ (*n* = 1)	ta_3_ (*n* = 1)
P_1_	976–1094, 1045	666–700, 688	–	–	–
P_2_	998–1100, 1047	752–872, 803	661	401	338
P_3_	938–1092, 1028	771–843, 810	537	239	182
	ta_4_ (*n* = 1)	ta_5_ (*n* = 1)	LR (*n* = 1)	BV (*n* = 1)	SV (*n* = 1)
P_1_	–	–	–	–	–
P_2_	184	105	0.84	2.4	2.8
P_3_	125	73	0.66	3.9	3.5

***Hypopygium*** (Fig. 6H). Tergite band present but short. Mid tergite IX with 5–6 long, simple setae, apex of tergite IX with rows of short, simple setae. Anal point absent. Sternapodeme 41–49, 46 μm long, tick, without oral projections. Phallapodeme 45–61, 53 μm long. Superior volsella 48–49 μm long, broadly pediform. Inferior volsella 75–96, 85 μm long, sub-cylindrical with a cluster of long apicoventral setae. Gonocoxite 85–98, 90 μm long. Gonostylus narrow 119–130, 123 μm long. HR 0.7–0.75, 0.73; HV 2.36–2.80, 2.59.

##### Etymology.

The new species is named in honor of Solangel and Jose Hugo Vasquez, the co-author’s parents, for their many years of support.

##### Remarks.

The new species is a very unusual *Lauterborniella*. Based on its absence of the anal point, at first glance, the species appears to belong to the genus *Apedilum*. However, the presence of fore tibia spur, separate tibial combs, presence of pulvilli, anal tergite band, median anal tergite setae, and rows of short apical setae on tergite IX places this species in *Lauterborniella*. The absence of the anal point and an unusually high antennal ratio distinguish *L. solangelhugo* sp. nov. from other known species of this genus.

#### Polypedilum (Tripodura) francisae sp. nov.

Taxon classificationAnimaliaDipteraChironomidae

D4DC6034-DFC4-596B-A632-FB7CECB20E19

https://zoobank.org/4C327E9B-0AAC-43CC-9314-9F5D2AF3B45B

Fig. 7

##### Type material.

***Holotype*** • 1 male; Belize, Guanacaste National Park, Roaring Creek, trib. to Belize River; 17.261937, -88.790266; leg. A.A. Vasquez; dep. ARC. ***Paratypes*** • 2 males; same as holotype; except one male with only the hypopygium.

##### Diagnostic characters.

The adult male of *P. francisae* sp. nov. can be separated from the related species by a combination of the following: AR 1.72–1.83; wing with a faint, large patch covering the basal portion of cell r_4+5_ and anteromedial portion of cell m_1+2_, faint patches also on the basal portion of cells m_3+4_, cu, and an; anal point broad and conoid with small basal notches; superior volsella robust, and tubular with a branch of long, basolateral, spine-like setae; gonostylus narrow and curved.

##### Description.

Male (*n* = 2; unless otherwise stated). Total length 3.07–3.15, 3.11 mm. Wing 1.57–1.61, 1.59 mm long and 0.46–0.47 mm wide. Total length/wing length 1.96. Wing length/profemur length 3.79–3.94, 3.87.

***Coloration***. Head, antenna, thorax, abdomen, and hypopygium brown. Wing pale brown with a faint patch covering the basal portion of cell r_4+5_ and anteromedial portion of cell m_1+2_, faint patches also on the basal portion of cells m_3+4_, cu, and an.

**Figure 7.**
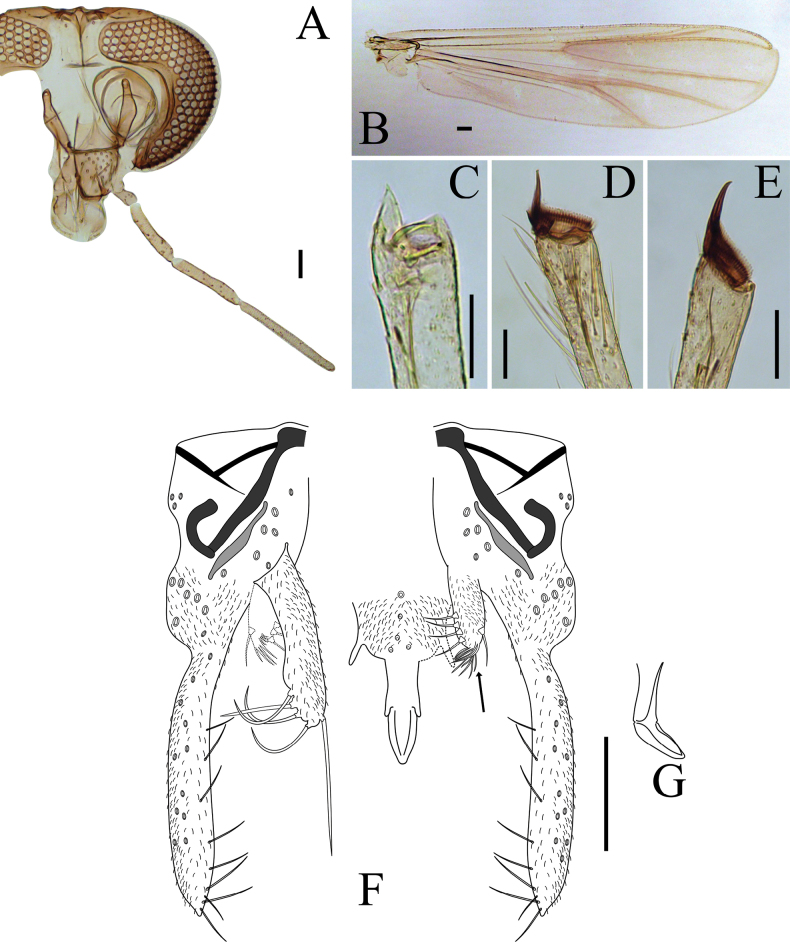
Polypedilum (Tripodura) francisae sp. nov. adult male. **A**. Head; **B**. Wing; **C**. Apex of fore tibia; **D**. Apex of mid tibia; **E**. Apex of hind tibia; **F**. Hypopygium, arrow indicates the superior volsella brush of long setae; **G**. Anal point, lateral view. Scale bars: 50 µm.

***Head*** (Fig. 7A). Antenna with 13 flagellomeres; 2^nd^-4^th^ flagellomeres each with a pair of long, thin sensilla chaetica as long as the segment bearing them; last flagellomere with 8 sensilla chaetica; AR 1.72–1.83, 1.78. Temporal setae 10, uniserial (*n* = 2). Clypeus nearly squared, 61–94, 78 μm long and 99 μm wide, bearing 20–26, 23 setae. Tentorium 146–163, 155 μm long. Length of palpomeres (in μm; 3–5 (*n* = 1)): 50–54, 52; 41–43, 42; 130; 150; 216.

***Thorax***. Acrostichals 20–21; dorsocentrals 15–16, uniserial; prealars 5; scutellars 10, uniserial. Antepronotal lobe well-separated, bare.

***Wing*** (Fig. 7B). Anal lobe well-reduced. R with 17–21, 19 setae; R_1_ with 16–19, 17 setae; R_4+5_ with 31–40, 35 setae. Brachiolum with 1 seta. Squama with 5–7 setae. VR 1.23–1.26, 1.25.

***Legs*** (Fig. 7C-E). Fore tarsus missing. Fore tibia with prominent triangular scale, 41–43 μm long. Mid tibia with divided comb and single spur, 67–72, 70 μm long. Hind tibia with divided comb and single spur, 53–63, 58 μm long. Lengths and proportions of legs as in Table 5.

**Table 5.** Male leg lengths (μm) and proportions of Polypedilum (Tripodura) francisae sp. nov.

	fe	ti	ta_1_ (*n* = 1)	ta_2_ (*n* = 1)	ta_3_ (*n* = 1)
P_1_	779–830, 804	507–559, 533	–	–	–
P_2_	853–878, 865	736–752, 744	443	249	185
P_3_	846–889, 867	813–889, 851	1087	655	497
	ta_4_ (*n* = 1)	ta_5_ (*n* = 1)	LR (*n* = 1)	BV (*n* = 1)	SV (*n* = 1)
P_1_	–	–	–	–	–
P_2_	109	85	0.59	3.3	3.68
P_3_	359	196	0.76	3.62	3.51

***Hypopygium*** (*n* = 3; Fig. 7F, G). Posterior tergite IX with lateral projections 21–26, 23 μm long. Anal point conoid with small basal notches, 59–76, 66 μm long, 23–24 μm wide at the base, and 15–22, 18 μm wide at the apex. Sternapodeme 53–62, 57 μm long. Phallapodeme 68–73, 71 μm long. Superior volsella 68 μm long, robust, and tubular with a branch of long, basolateral, spine-like setae. Inferior volsella 117–132, 126 μm long, sub-cylindrical with a cluster of long apicoventral setae. Gonocoxite 100–109, 105 μm long. Gonostylus narrow and curved, 185–195, 191 μm long. HR 0.54–0.56; HV 1.62–1.66, 1.64.

##### Etymology.

The new species is named in honor of Francis Vasquez, the co-author’s wife, for her many years of support.

##### Remarks.

**Figure 8.**
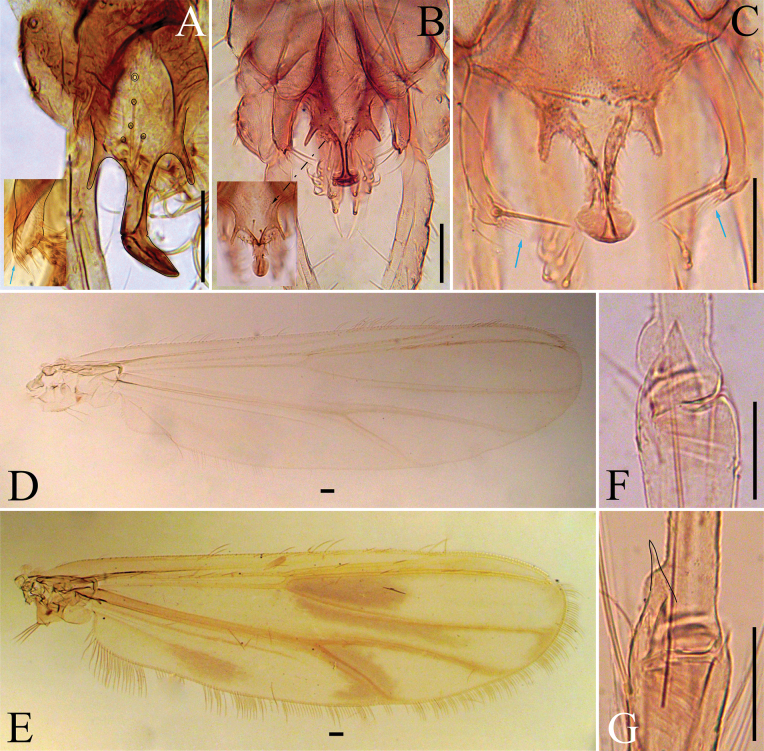
*Polypedilum* Kieffer, 1912 adult males. **A**. Polypedilum (Tripodura) francisae sp. nov.; **B, D, F**. Polypedilum (Tripodura) digitifer Townes, 1945; **C, E, G**. Polypedilum (Tripodura) griseopunctatum (Malloch, 1915); **A–G**. Hypopygium, blue arrows indicate the superior volsella brush; **D, E**. Wing; **F, G**. Fore tibia scale. Scale bars: 50 µm.

*P. francisae* sp. nov. closely resembles the Nearctic species Polypedilum (Tripodura) digitifer Townes, 1945, and Polypedilum (Tripodura) griseopunctatum (Malloch, 1915). The tergite IX of *P. digitifer* has numerous setae, particularly adjacent to the anal point. The tergite IX of *P. francisae* sp. nov. and *P. griseopunctatum* have only a few setae with no long setae adjacent to the base of their anal point. The anal point of *P. francisae* sp. nov. is longer on average, 66 μm, and narrower, 23–24 μm at the base and 15–22 μm at the apex. Soponis and Simpson (1992) did not provide any measurements for the anal points of *P. digitifer* and *P. griseopunctatum*. However, based on examination of the adult males of these species from Alabama, USA, both species have on average shorter (53–59, 56 μm & 57 μm, respectively) and wider anal points (33–40, 37 & 33 μm at base; 21–28, 23 & 28 μm at apex, respectively). The apex of *P. francisae* sp. nov. anal point is conoid, whereas *P. digitifer* and *P. griseopunctatum* are crescent-shaped. *P. griseopunctatum* anal point is setose with numerous minute lateral setae, whereas those of *P. digitifer* and *P. francisae* sp. nov. are bare (Fig. 8A–C). The superior volsella of the new species has a branch of long, basolateral, spine-like setae, which is only present basally on *P. griseopunctatum* superior volsella. However, species differ in the shape and size of their superior volsella (Fig. 8A–C). *P. griseopunctatum* superior volsella is 70–74 μm long; whereas those of *P. digitifer* and *P. francisae* sp. nov. are 47–65 μm and 68 μm long, respectively. *Polypedilum digitifer* wing has no markings, whereas *P. francisae* sp. nov. and *P. griseopunctatum* have markings, albeit those of *P. griseopunctatum* are more prominent (Figs 7B, 8D, 8E). The fore tibia scales of *P. digitifer* and *P. francisae* sp. nov. are wider with short spinous apex, whereas *P. griseopunctatum* fore tibia scale is narrower and has a long spinous apex (Figs 7C, 8F, 8G). Among the Neotropical species, the apex of the anal point of the new species resembles those of Polypedilum (Tripodura) epomis Sublette & Sasa, 1994, Polypedilum (Tripodura) luteopedis Sublette & Sasa, 1994, Polypedilum (Tripodura) titicacae Roback & Coffman, 1983, Polypedilum (Tripodura) bacalar Vinogradova, 2008, and Polypedilum (Tripodura) scharfi Vinogradova, 2008. However, the latter species lack a basal notch on their anal points. Furthermore, their posterior tergite IX lateral projections are much shorter compared to the new species. Among the Neotropical species, *P. titicacae* and Polypedilum (Tripodura) umayo Roback & Coffman, 1983 have superior volsella with spine-like setae. However, the latter species, superior volsellae, differ in shape and size from the new species.

Based on Bidawid and Fittkau’s (1995) categorizations of the morphological characters of the Neotropical *Polypedilum*, the new species is characterized as in Table 6.

**Table 6.** Morphological characters for Polypedilum (Tripodura) francisae sp. nov. based on Bidawid and Fittkau (1995).

Morphological character	Type
Mid and hind leg tibia spur and comb	Type A
Fore tibia scale	Type B
Median tergite band	Type F
Superior volsella	Type B_1_
Inferior volsella	Type D
Gonostylus	Type B

The genus is under revision for the Neotropical realm. The faunistic records, descriptions, and major studies on the Neotropical species of *Polypedilum* can be found in the works of Armitage et al. (2025), Ballesteros et al. (2022), Baranov et al. (2020), Bello-González et al. (2024), da Silva et al. (2023), Pinho and da Silva (2020), Pinho et al. (2015), Bidawid and Fittkau (1995), Bidawid-Kafka (1996), Fuentes and Donato (2014), Mendes et al. (2011), Roback and Coffman (1983), Shimabukuro et al. (2019), Sublette and Sasa (1994), and Vinogradova (2008).

## Supplementary Material

XML Treatment for Labrundinia belizensis

XML Treatment for Cricotopus mayaorum

XML Treatment for Axarus rogersi

XML Treatment for Lauterborniella solangelhugo

XML Treatment for Polypedilum (Tripodura) francisae

## Additional information

### Use of AI

No use of AI was reported.

### Author ORCIDs

Armin Namayandeh https://orcid.org/0000-0003-2136-0497

Adrian A. Vasquez https://orcid.org/0000-0002-2434-3388

### Data availability

All of the data that support the findings of this study are available in the main text.
